# Audit of the Assessment and Documentation of Sexual Side Effects of Antipsychotic Medication in an Inpatient Rehabilitation Ward

**DOI:** 10.1192/bjo.2026.11695

**Published:** 2026-06-30

**Authors:** Gabriela Paduret, Mohammed Al-Dabbagh, David Ibrahim, Hanif Soomro

**Affiliations:** Northamptonshire Healthcare NHS Foundation Trust, Northampton, United Kingdom

## Abstract

**Aims:**

Sexual dysfunction (SD) is a common side effect of antipsychotic treatment, affecting desire, arousal, orgasm, and sexual pain, and may reduce quality of life and medication adherence. Despite this, it is often overlooked in clinical settings. NICE CG178 recommends annual review of antipsychotic side effects, including sexual side effects. Improving the management of sexual side effects has the potential to enhance both quality of life and medication adherence. This audit aimed to establish a baseline understanding of the documentation of sexual side effects in patients receiving antipsychotics in Meadowbank Ward (a 12-bed inpatient specialist adult male rehabilitation ward in Berrywood Hospital), assess adherence to NICE CG178 guidelines and identify areas for improvement.

**Methods:**

A retrospective case notes review of all the patients admitted to Meadowbank Ward between 1^st^ of January 2023 and 1^st^ of January 2026. The collected data included age at discharge, length of admission to the ward, ethnicity, primary diagnosis, documentation of sexual side effects within the last 12 months of their admission, and subsequent actions.

**Results:**

Twenty-two male patients met the inclusion criteria. Age at discharged ranged between 21-47 years-old. Primary diagnoses were schizophrenia (n=18, 82%), schizoaffective disorder (n=3, 14%) and delusional disorder (n=1=5%). Three patients (14%)had neurodevelopmental comorbidity (ASD or ADHD). Documentation of sexual side-effect discussion within 12 months was identified for 18 patients (82%). No patients declined discussion. Nursing staff authored for 14/18 patients in the trust’s physical health monitoring and intervention template and medical staff documented entries for 4/18 patients in ward round notes. Four patients (22%) of those with documented discussions reported sexual side effects. Actions included physical health check with blood tests (n=1), reduction of antipsychotic dose (n=1), use of a herbal remedy (n=1), and no documented action (n=1).

**Conclusion:**

This audit demonstrates that most patients (82%) had documented discussion regarding sexual side effects of antipsychotic medication, with nursing staff effectively utilising the Trust’s monitoring template. However, the audit identified incomplete adherence to NICE CG178 standard (target of 100%) and inconsistent clinical responses when SD was reported. The plan includes conducting a staff survey to identify barriers to discussion and documentation of sexual side effects; developing a standardized pathway for the assessment and management of antipsychotic-related SD; providing targeted training for medical and nursing staff; presenting findings to the MDT and Trust’s audit committee; and conducting a re-audit.

